# Updates in CT characterization of thymic epithelial tumors in patients with myasthenia gravis

**Published:** 2012

**Authors:** GA Popa, V Tomulescu, I Popescu, V Herlea, IG Lupescu

**Affiliations:** * Department of Radiology and Medical Imaging, Fundeni Clinical Institute, Bucharest, Romania; ** Department of General Surgery and Liver Transplantation, Fundeni Clinical Institute, Bucharest, Romania; *** Department of Pathology, Fundeni Clinical Institute, Bucharest, Romania

**Keywords:** Thymus, myasthenia gravis, computed tomography, myasthenia gravis = MG, computed tomography = CT

## Abstract

Thymic epithelial tumors have been traditionally classified into two main types: noninvasive and invasive thymoma. Several classifications have been proposed for thymic tumors, but according to these classifications, the prognosis of patients with thymomas varies considerably. Our purpose is to present different CT aspects according to various subtypes of thymic epithelial neoplasms based on the simplified World Health Organization classification. In this article, we will discuss and illustrate histologic and functional features of the thymus and a spectrum of thymic tumors associated with Myasthenia Gravis. Smooth contours and a round shape are the most suggestive of type A thymic epithelial tumors, whereas irregular contours and heterogeneous enhancement are the most suggestive of type C tumors. Calcifications are suggestive of type B tumors. CT findings may serve as predictors of postoperative recurrence or metastasis for the thymic epithelial tumors.

**Purpose.** In this article, we will discuss and illustrate histologic and functional features of the thymus and a spectrum of thymic tumors associated with Myasthenia Gravis (MG). We considered the current World Health Organization (WHO) histologic classification scheme for thymic epithelial tumors (**Table 1**).

**Table 1.** Morphological aspects of thymomas (after WHO 2004)

**Type**	**Morphological aspects of thymomas**
**Type A**	A tumor composed of neoplastic epithelial cells, spindle or oval in shape, inconspicuous nucleoli, without nuclear atypia, and few or no lymphocytes.
**Type AB:**	A tumor, which consists of areas similar to those from A thymoma, but mixed with lymphocyte-rich areas, the border between being sharp or less distinct.
**Type B1**	The tumor resembling the typical thymus appearance, associating areas similar to the thymic cortex with foci with medullary differentiation. The cortical areas are prevalent and in excess compared to the small medullary areas. The neoplastic epithelial cells are scant, small and dispersed in the lymphocytic component.
**Type B2**	A tumor composed of large plump/polygonal neoplastic epithelial cells, with vesicular nuclei and distinct nucleoli, the tumoral cells are usually outnumbered by the non-neoplastic lymphocytes. The perivascular spaces are common.
**Type B3**	Tumor composed predominantly of round/polygonal neoplastic epithelial cells; the nucleoli are less prominent, with mild nuclear atypia and with a poor lymphocytic component. The perivascular spaces and squamous metaplasia are common.
**Thymic Carcinoma**	Thymic carcinoma: Thymic tumor with loss of organotipical differentiation of the organ and with clear cytological atypia, generally similar to that encountered in other organs. There is a lymphocyte population, which is mature.

**Anatomic, Histologic and Functional Features of the Thymus.** The thymus is a heterogeneous admixture of lymphoid and epithelial elements, located in the superior and anterior portions of the mediastinum. The thymic shape is partially determined by adjacent structures (**Fig. 1**).

**Fig.1:**
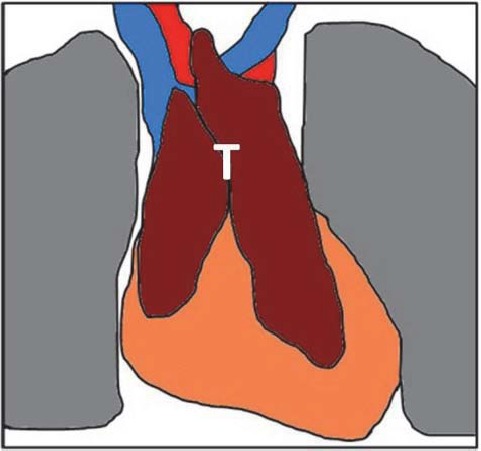
The thymus of an adolescent (T), ventral view (anatomic drawing)

The thymus achieves its maximal weight between 12 and 19 years; between 20 and 60 years, regression in size occurs, together with the replacement by adipose [**1**]. A thin connective tissue capsule surrounds each lobe and gives rise to septae, that partially subdivide the thymus into interconnecting lobules of variable size and orientation [**2**]. The cortex is composed primarily of lymphocytes (thymocytes), with a few epithelial and mesenchymal cells. The medulla is mainly composed of epithelial cells. The epithelial cells are functionally essential for the maturation of T lymphocytes and thus are called “nurse cells” [**3**]. Hassall corpuscles are the characteristic structures of the thymus and are found exclusively in the medulla [**3**]. These entities are epithelial cells in the thymic medulla that generally have a large nucleus, degenerative changes in the cytoplasm, and cytoplasmic keratinization [**2**]. In addition to epithelial cells and lymphocytes, the thymus contains a variety of other types of cells, including macrophages and myoid cells; myoid cells have an important role in the pathogenesis of MG [**3**]. They have ultrastructural and immunohistochemical features of striated muscle [**2**]. The thymus is a primary lymphoid organ; bone marrow derived progenitor cells undergo differentiation/maturation, within the thymic microenvironment, to form the functional T cell repertoire [**2**].

**CT technique.** The scanning of the mediastinum is usually done as part of a general thoracic CT examination. Scans were obtained in inspiration with the patient in the supine position. Contiguous 1-0,5cm collimation slices are performed throughout the mediastinum. Contiguous 0,3-0,5cm collimation slices are performed for densitometric characterization of micronodules located in the anterior mediastinum. Intravenous administration of nonionic iodinated contrast media is used occasionally, in selected patients, to delineate thymus from the aorta, superior vena cava and pulmonary artery. Thick coronal or sagittal reformations improve the evaluation of the thymic masses. Thin-slab MIP (maximum intensity projection) or VRT (volume rendering technique) can help to define vascular structures.

**CT features of thymic tumors.** Thymomas are generally seen as homogeneous, soft-tissue masses located in the anterior mediastinum, usually projects to one side of the mediastinum, and can have well-demarcated or lobulated borders. On CT obtained after intravenous administration of contrast material, the mass enhances homogeneously, unless necrosis and hemorrhage are present [**4**]. Areas of decreased attenuation corresponding to cystic changes; the attenuation values of the cyst fluid may approach those of soft tissue, depending on the composition of the fluid**.** Calcification within a thymoma may be detected on plain radiographs. The pattern of calcifications is commonly linear, thin, and peripheral and corresponds to calcium deposition in the tumor capsule. Calcified foci may also be seen scattered throughout the tumor [**4,5**]. CT demonstrates tumoral extension to the surrounding mediastinal fat, vascular structures and the adjacent lung. Fatty involution of the thymus makes the detection of thymoma easier in patients over 40 years of age [**4,5**].

**CT Findings of Thymic Epithelial Tumors Based on the simplified WHO.** Following CT aspects should be evaluated: contour (smooth, lobulated, irregular), shape (round, oval, plaque), area of necrosis, calcification, enhancement pattern (homogeneous, heterogeneous), enhancement degree, mediastinal fat invasion, great vessel invasion, pleural effusion, pericardial effusion, pleural seeding, pericardial seeding, lymph node metastasis and hematogenous metastasis. Jeong et al. simplified the WHO histologic classification scheme (**Table 2**) into three subgroups: low risk thymomas (types A, AB, and B1), high-risk thymomas (types B2 and B3), and thymic carcinomas, and correlated CT findings in the three tumor subgroups with prognosis [**3,6**].

**Table 2.** Simplified WHO Classification of Thymic Epithelial Tumors

**Simplified WHO Classification**		
**Low-Risk Thymoma**	**High-Risk Thymoma**	**Thymic Carcinoma**
A, AB, B1	B2, B3	C

CT aspects are grouped in **Table 3**, according to the simplified WHO (2004) classification of the thymic epithelial tumors. The CT findings of the thymic epithelial tumors have many degrees of overlap between subgroups of the simplified WHO classification [**6-8**].

**Table 3.** CT Findings of Thymic Epithelial Tumors Based on the Simplified WHO Classification (adapted from Jeong et al.)

**CT Findings of Thymic Epithelial Tumors Based on the WHO Classification.** **Adapted from Jeong et al.**				
**CT Finding**		**Simplified WHO Classification**		
		**Low-Risk Thymoma**	**High-Risk Thymoma**	**Thymic Carcinoma**
**Contour**	**Smooth**	**+++**	**++**	**-**
	**Lobulated**	**+**	**++**	**+**
	**Irregular**	**-**	**+**	**++**
**Shape**	**Round**	**+**	**++**	**±**
	**Oval**	**+**	**+++**	**±**
	**Plaque**	**-**	**+**	**-**
**Area of necrosis**	**Absence**	**+++**	**++**	**+**
	**Presence**	**-**	**++**	**++**
**Calcification**		**-**	**++**	**±**
**Enhancement pattern**	**Homogeneous**	**++**	**++**	**+**
	**Heterogeneous**	**+**	**++**	**++**
**Enhancement degree**	**Less**	**+**	**+**	**-**
	**Equal**	**+++**	**+++**	**++**
	**More**	**++**	**++**	**±**
**Mediastinal fat invasion**		**-**	**+**	**++**
**Great vessel invasion**		**-**	**-**	**++**
**Pleural effusion**		**-**	**++**	**++**
**Pericardial effusion**		**-**	**++**	**++**
**Pleural seeding**		**-**	**++**	**+++**
**Lymph node metastasis**		**-**	**++**	**+**

Tomiyama et al. assessed the CT features of various subtypes of thymic epithelial tumors in 53 patients and reported that smooth contours and a round shape are most suggestive of type A tumor, irregular contours and mediastinal lymphadenopathy are most suggestive of type C tumor, and calcification is suggestive of type B1, B2, and B3 tumors [**3,7**].

The combination of homogeneous enhancement and a high degree of enhancement is suggestive of type A and AB tumors; heterogeneous enhancement is seen more often in types B3 and C [**7**]. In the studies of Tomiyama et al. mediastinal lymphadenopathy is present in 43% of type C tumors, 7% of type AB, but not in other types of thymic epithelial tumors [**7**]. Do et al. reported that mediastinal lymphadenopathy was present in 40% of thymic carcinomas (type-C thymoma) and 8% of invasive thymomas [**9**]. Jeong et al. reported that lymphadenopathy was present in 13% of type C tumors; selection bias may have contributed to these results [**6**].

According to Tomiyama et al. and Jung et al., type C tumors were significantly larger than any other type of thymic epithelial tumors [**7,10**]. In the study of Jeong et al. the long- and short-axis diameters of thymic carcinoma (type C tumors) were larger than those of low- and high-risk thymomas, but the statistically significant difference was present only between high-risk thymomas and thymic carcinomas [**6,8**]. Invasion of the great vessels, mediastinal lymphadenopathy, extrathymic metastases and phrenic nerve palsy occur only in patients with thymic carcinoma [**8,10**]. Thymic carcinomas are less commonly associated with pleural implants than invasive thymoma [**8,9**]. In the study of Sadohara et al., irregular contours, necrotic or cystic component, heterogeneous contrast-enhancement, lymphadenopathy, and great vessel invasion were more commonly seen in thymic carcinomas than low- and high-risk thymomas on CT [**11**].

Of many CT findings of thymic epithelial tumors, only the contour of tumors, mediastinal fat, and great vessel invasion enabled us to differentiate subgroups of the simplified WHO classification. Tumors with a lobulated or irregular contour, oval shape, mediastinal fat or great vessel invasion, or pleural seeding show significantly high recurrence and metastasis rates. Okumura et al. reported that type B2 and B3 tumors had more malignant nature in terms of prognosis and tumor recurrence than types A, AB, and B1 tumors [**11,12**]. Type C tumors apparently had a much more aggressive nature and indicated a poorer prognosis compared with other types of tumors, and they seemed to constitute a group of tumors oncologically distinct from type A, AB, B1, B2, and B3 tumors. Type C tumors comprise several subtypes of thymic carcinomas with distinct aggressiveness. Patients with type C tumors were excluded from this study [**12**]. Although the CT has a limited value in differentiating the histologic subtypes according to the WHO classification, CT findings may serve as predictors of postoperative recurrence or metastasis for this tumor type [**10,13**] (**Fig. 2****-****5**).

**Fig. 2:**
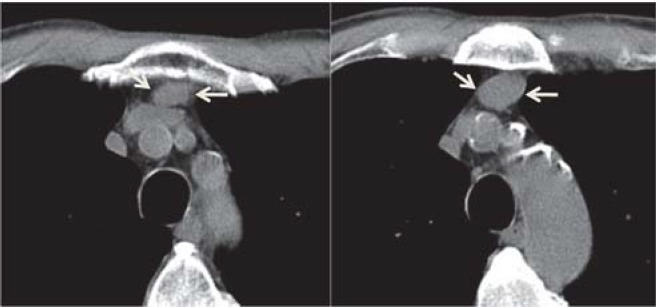
66-y/o male with myasthenia gravis; CT scan shows homogeneous anterior mediastinal mass with round-ovalar shape and smooth contours (arrows); pathologic diagnosis - type A thymoma

**Fig. 3:**
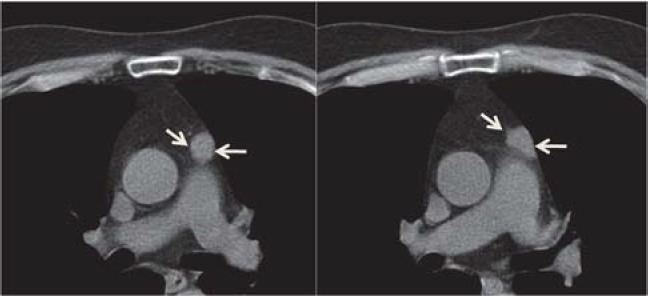
42-y/o male with myasthenia gravis; CT scan shows tumoral nodule in the anterior medistinum (arrows); pathologic diagnosis - type AB thymoma

**Fig. 4:**
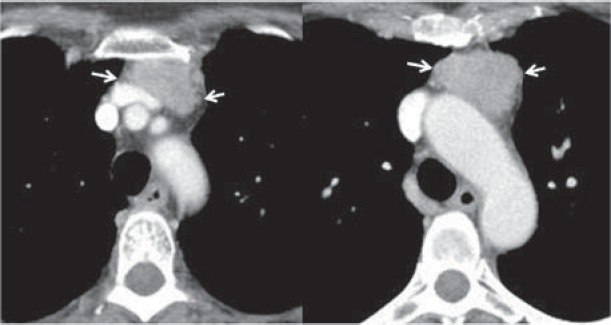
64-y/o female with myasthenia gravis; Contrast-enhanced CT scan shows slightly heterogeneous anterior mediastinal mass with irregular contour (arrows); pathologic diagnosis - type B3 thymoma

**Fig. 5:**
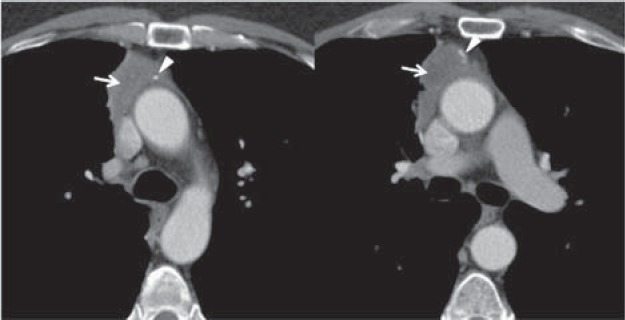
52-y/o male with myasthenia gravis; Contrast-enhanced CT scan shows lobulated contour mass at the anterior mediastinum with heterogeneous enhancement, areas of decreased attenuation (arrows) and calcified foci (arrowheads); pathologic diagnosis – type B3 thymoma.

**Differential diagnosis.** Differential diagnosis of thymic masses is performed mainly with malignant lymphoma, lymph nodes enlargement and germ cell tumors. Sometimes, aberrant parathyroid or thyroid tissue masses are found. Neoplasms and other masses originating from vascular or mesenchymal tissues also occur in anterior mediastinum. Age is a significant factor in the differential diagnosis of mediastinal masses. Malignant lymphoma, benign thymic enlargement and germ cell tumors represent the main lesions in the anterior mediastinum in children.

**Take home messages.** CT can be useful in differentiating non-invasive from invasive thymomas, but has a limited value in distinguishing thymomas from lymphoid follicular hyperplasia. Smooth contours and a round shape are most suggestive of type A thymic epithelial tumors, whereas irregular contours and heterogeneous enhancement are most suggestive of type C tumors. Calcifications are suggestive of type B tumors. Although CT has limited value in differentiating histologic subtypes according to the WHO classification, CT findings may serve as predictors of postoperative recurrence or metastasis for the thymic epithelial tumors.
